# Highly Sensitive Detection of PCV2 Based on Tyramide Signals and GNPL Amplification

**DOI:** 10.3390/molecules24234364

**Published:** 2019-11-29

**Authors:** Shouping Zhang, Bin Hu, Xiaojing Xia, Yanzhao Xu, Bolin Hang, Jinqing Jiang, Jianhe Hu

**Affiliations:** College of Animal Science and Veterinary Medicine, Henan Institute of Science and Technology, Eastern HuaLan Avenue, Xinxiang 453003, China; spzhang@hist.edu.cn (S.Z.); hubin24kb@163.com (B.H.); xjxia@hist.edu.cn (X.X.); xuyanzhao@hist.edu.cn (Y.X.); yzhbl001@126.com (B.H.); jjq5678@126.com (J.J.)

**Keywords:** gold nanoparticle, PCV2, sensitive, amplification, tyramide

## Abstract

The frequent emergence of secondary infection and immunosuppression after porcine circovirus type 2 (PCV2) infection highlights the need to develop sensitive detection methods. A dual-signal amplification enzyme-linked immunosorbent assay (ELISA) based on a microplate coated with gold nanoparticle layers (GNPL) and tyramide signal amplification (TSA) was established. Results confirmed that the microplates coated with GNPL have a strong binding ability to the antibody without affecting the biological activity of the antibody. The microplates coated with GNPL have strong binding ability to the antibody, and the amplification of the tyramide signal is combined to further improve the sensitivity of PCV2. The PCV2 antibody does not crossreact with other viruses, demonstrating that the method has good specificity. A dual-signal amplification strategy is developed using microplates modified with GNPL and TSA to sensitively detect PCV2.

## 1. Introduction

Porcine circovirus type 2 (PCV2), which belongs to the genus *Circovirus* in the family *Circoviridae*, has been identified as the major etiological agent of postweaning multisystemic wasting syndrome (PMWS). With the development of China’s swine industry, diseases affecting pigs have become increasing complicated in swine herds, and PCV2 tends to present more complicated symptoms [1]. PCV2 not only causes primary infection in pigs, but also damages the immune system, thereby causing infection by other bacteria or viruses (*Streptococcus suis*, porcine reproductive and respiratory syndrome virus, porcine parvovirus, pseudorabies) and coinfection or secondary infections [2]. Unfortunately, only a subset of infected pigs presents the above symptoms after infection; hence, most infected pigs belong to the subclinical infection without any clinical manifestations. In 2016, PCV3, a novel porcine circovirus, was identified and it has clinical symptoms similar to that of PCV2 [3,4]. Thus, developing highly sensitive and reliable methods for PCV2 detection at an early stage is important.

A number of methods have been developed for detecting PCV2. For example, an SYBR green or TaqMan probe-based real-time PCR has been established. In addition, a more sensitive droplet digital PCR (ddPCR) was applied to detect PCV2 and PCV3 [5,6,7]. These methods facilitate the epidemiological investigations of PCV2. However, PCR requires complex equipment, such as a thermocycler, real-time PCR, Gelsystem, and Gel imager, thereby limiting its wide use. Enzyme-linked immunosorbent assay (ELISA) is one of the most widely used analytical techniques in clinical medicine because of its adaptability, simplicity, and sensitivity [8]. Gold nanoparticles (AuNPs), which are also widely used in biomedicine, have a high specific surface area and unique physicochemical properties [9,10]. Gold nanoparticles can easily combine antibodies, enzymes, nucleotide sequence, and other biomolecules without changing their characteristics. Many studies have demonstrated that gold nanoparticles have a signal amplification effect on biochemical detection. ELISA modified with gold nanoparticle layers (GNPL) are more sensitive than conventional commercialized ELISA kits in carcinoembryonic antigen detection [11,12].

Tyramide signal amplification (TSA) is a horseradish peroxidase (HRP)-mediated signal amplification method. In the assay, HRP catalyzes the deposition of tyramide conjugates, such a biotinyl-tyramide, on a solid phase. A subsequent reaction with streptavidin HRP results in the localization of the enhancement of the HRP signal at the site of tyramide deposition [13,14]. TSA has been widely used in ELISA, flow cytometry, immunohistochemistry, and in situ hybridization for the detection proteins, DNA, and pathogens because of its excellent amplification effects and simple operations. A 100-fold sensitivity improvement was achieved by in situ hybridization by using biotin-labelled oligodeoxynucleotides and tyramide signal amplification in foot and mouth disease virus (FMDV) detection [15]. A study developed a highly sensitive in situ hybridization method following the tyramide signal amplification method that can detect porcine reproductive and respiratory syndrome (PRRSV) in paraffin-embedded tissues [16].

A dual amplification strategy was constructed by combining TSA- and GNPL-coated microplates to develop a simple and sensitive ELISA method for PCV2 detection. The results showed that this method could be used to sensitively quantify PCV2, and possibly detect low PCV2 levels from the samples. To the best of our knowledge, this study is the first report on the highly sensitive detection of PCV2.

## 2. Results and Discussion

### 2.1. Basic Principles of the Dual Signal Amplification-Based Detection Method

**Figure 1:**
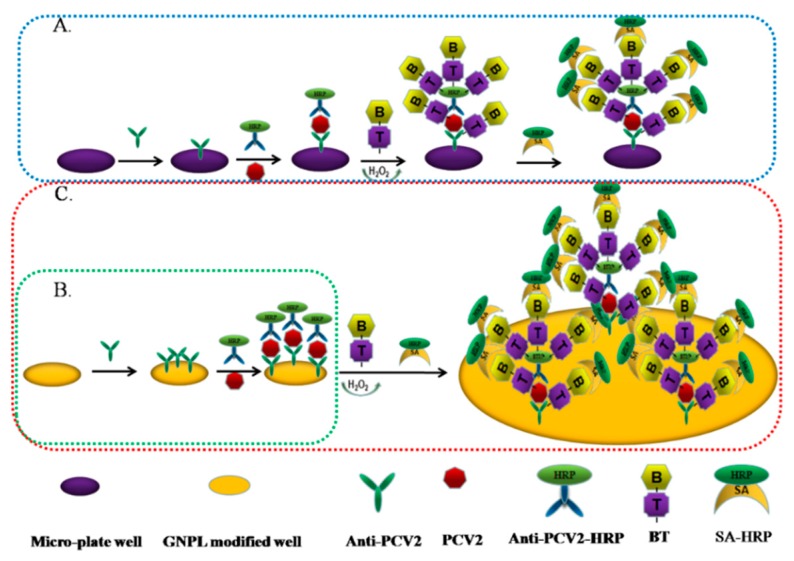
The working principle of the dual signal amplification enzyme-linked immunosorbent assay (ELISA) based on gold nanoparticle layers (GNPLs) and tyramide for the sensitive detection of Porcine circovirus type 2 (PCV2). (**A**): Separate tyramine signal amplification (TSA) technique; (**B**): Separate GNPL amplification technique; (**C**) dual signal amplification (GNPL + TSA)-based ELISA.

GNPL-modified microplates can absorb more capture antibodies (anti-PCV2), followed by more PCV2 antigen binding, and then form a sandwich through antigen-binding anti-PCV2-HRP antibodies. Tyramine signal amplification is performed to deposit a large amount of HPR, catalyze the TMB color development of the substrate, and sensitivity detect PCV2. The principle of the dual signal amplification detection principle is shown in Figure 1 as the red dotted line. A separate TSA technique, shown by the blue dotted line in Figure 1A, and a separate GNPL modified microplate, shown by the green dotted line in Figure 1B, were used as controls.

### 2.2. Verification of the Effect of Dual Signal Amplification

**Figure 2:**
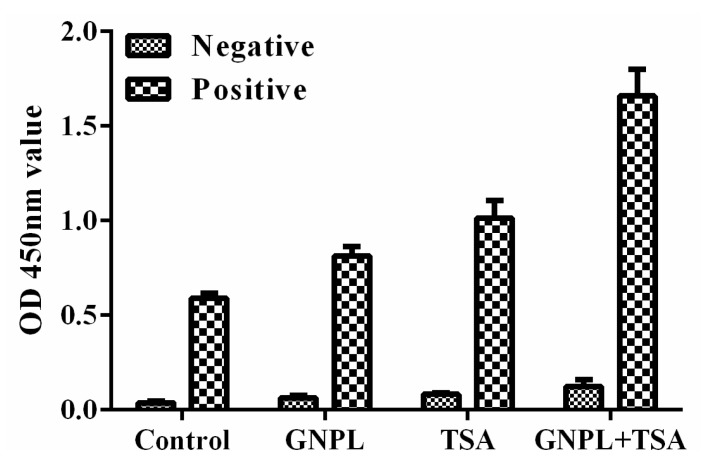
Verification of enhanced sandwich immunoassay for PCV2 detection based on GNPL and TSA. Experiment conditions: GNPL solution: 60 µL; anti-PCV2 antibody: 10 μg/mL; anti-RCV2-HRP: 1 µg/mL; BT: 10 µL/mL; SA-HRP: 1/500 dilution; PCV2: 20TCID_50._

The unamplified (normal double antibody sandwich ELISA), separate TSA signal amplification, separate GNPL signal amplification, and dual amplification (TSA + GNPL) methods were constructed separately to verify the efficiency of dual signal amplification for PCV2 detection. The PCV2-positive sample displayed a markedly enhanced signal through the dual signal (TSA + GNPL) amplification method compared to that of the unamplified method, whereas the signal through the separate TSA and GNPL amplification method was limited. The control sample (PCV2-negative sample) displayed a low signal through these four detection methods, indicating that the nonspecific adsorption signal was effectively blocked by BSA, as seen in Figure 2. These results demonstrated that the dual signal amplification strategy is feasible.

### 2.3. Characterization of GNPL

**Figure 3:**
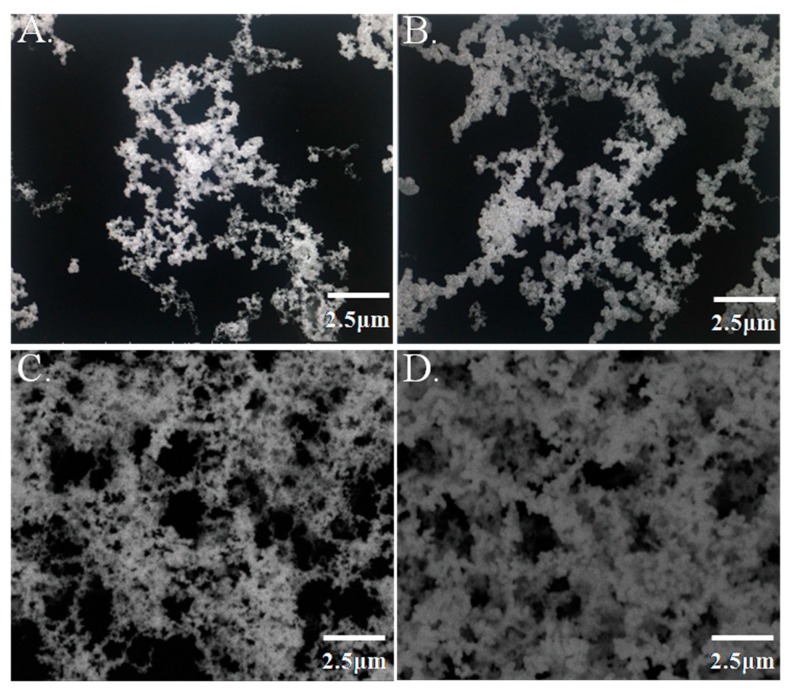
Characteristic images of gold nanolayer-modified microplates Different volumes (**A**) 10 μL, (**B**) 30 μL, (**C**) 60 μL, (**D**) 90 μL of electroplating solution were added to the microplate well, and the characteristics of the GNPL on the microplate were obtained using a Hitachi S-4800 scanning electron microscope (Tokyo, Japan) at 20.0 kV under 10,000 × magnification.

Immunoassay results are closely related to the number of capture antibodies. The higher the number of capture antibodies adsorbed in the microplates, the better the detection performance will be. In our research, GNPL-modified microplates were prepared through electroless plating. An electrofluid with different volumes (10 µL, 30 µL, 60 µL, and 90 µL) was added to the microplate wells to examine the effect of GNPL on the detection performance. The morphological characteristics of these series GNPL-modified microplates were observed through scanning electron microscopy, and porosity was analyzed using Image J. A large number of nanoparticles with size of about 150–200 nm accumulated on the surface of the inner well of the microplate and formed the GNPL. As the volume of the electrofluid increased, the number of gold nanoparticles in the well gradually increased, and the gap between particles considerably decreased, as seen in Figure 3. However, light transmittance was also affected as the volume increased. Thus, in our study, 90 μL was selected as the optimal volume of the modified GNPL.

Gold nanoparticles have been widely explored because of their high surface-to-volume ratio, high loading capacity, and long-term stability. In our study, gold nanoparticles were electroplated in the microplate and formed a gold nanoparticle layer to maximize the effectiveness of the loading capacity. The SEM data showed that the GNPL significantly increased the surface area, allowing the nanoparticles to absorb more PCV2 antibodies. A previous study reported that the sandwich ELISA-based GNPL-coated microplate markedly amplified the representative cancer biomarker CEA detection signal, exhibiting higher sensitivity than the commercialized CEA ELISA kit [17].

### 2.4. Establishment and Evaluation of the Dual Signal Amplification-Based Detection Method

**Figure 4:**
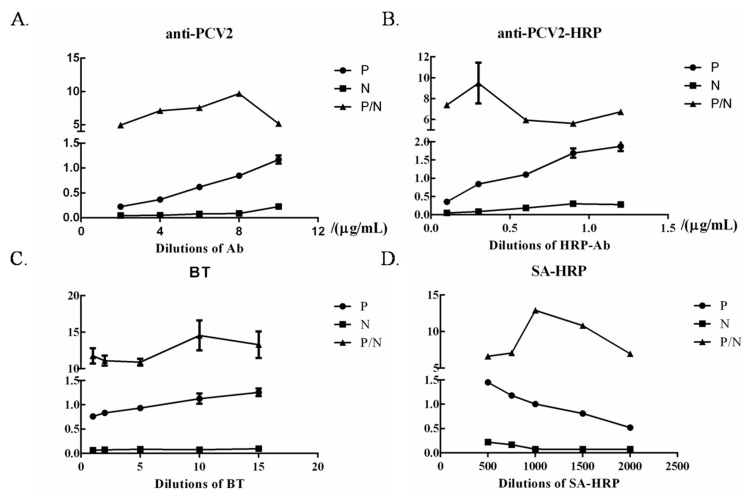
Optimization of dual signal amplification ELISA working conditions. The optimal anti-PCV2 (**A**) and anti-PCV2-HRP dilutions (**B**), dilutions of the SA (**C**), and the dilution of SA-HRP (**D**) were determined through checkerboard titration.

The concentration of anti-PCV2 and anti-PCV2-HRP is an important factor affecting the detection assay sensitivity. BT and SA-HRP also affect the sensitivity. The optimal dilution concentration of each indicator was determined through titration. The optimal dilution concentration of anti-PCV2, anti-PCV2-HRPand BT were 8 μg/mL, 0.3 μg/mL, and 10 μL/mL respectively, as seen in Figure 4. The optimal dilution concentration of SA was 1:1000. At these concentrations, the OD 450 nm value was close to 1.0, and the P/N was at the maximum.

The negative samples collected in the laboratory were statistically analyzed with this method. The average was X = 0.096, the standard deviation was 0.0087, and X + 2 × SD = 0.113. Therefore, the sample with OD450 < 0.113 was identified as negative in this method; otherwise, it was identified as a positive sample.

**Figure 5:**
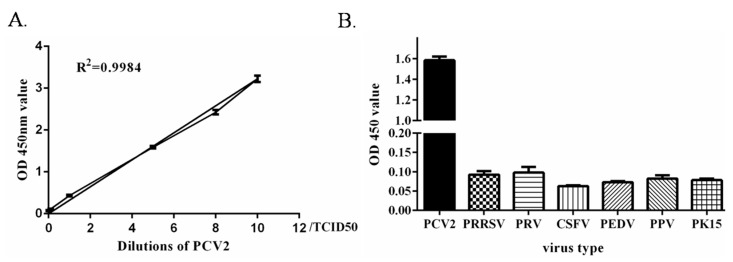
Standard curve and specificity of dual signal amplification ELISA. (**A**) Standard curve of the dual signal amplification ELISA. The high concentration of the PCV2 sample was diluted into a series of standards (10 TCID50, 8 TCID50, 5 TCID50, 1 TCID50, 0.1 TCID50, and 0.05 TCID50) for ELISA test. (**B**) The specificity of dual signal amplification. The PCV2, PRRSV, PRV, CSFV, PEDV, PPV, and PK15 were diluted to 5 TCID50 for ELISA testing, respectively.

Through this method, the limit of detection was 0.1 TCID50 and linearity was good (R^2^ = 0.99), as seen in Figure 5A. The specificity test results showed that the absorbance values of PRRSV, PRV, CSFV, PEDV, and PPV were less than 0.113, as seen in Figure 5B, indicating that the PCV2 antibody did not crossreact with other viruses. This result demonstrated that the method had excellent specificity. Three positive samples were tested at different times and under the same conditions. Each sample was repeated three times, and the coefficient of variation of the OD 450 nm value was calculated. The results in Table 1 showed that the intra-assay coefficient of variation and interassay coefficient of variation of the three samples were between 4.25% and 9.46%; both less than 10%, indicating that the method was reproducible.

The TSA signal detection system has been developed and primarily used or detection of low copy number mRNA through fluorescent in situ hybridization. TSA is also used to enhance the low expression levels of protein signals through immunohistochemistry. However, when used in detecting proteins, biotinyl-tyramide produces a strong nonspecific signal that compromises the amplification effect [18]. Moreover, endogenous peroxidases can catalyze the biotinyl-tyramide reaction, which may be responsible for background issues in protein detection, especially in cell samples. In our study, the concentrations of primary and secondary antibodies, and BT, SA-HRP induced a high nonspecific signal. The concentrations of the antibodies and TSA were optimized to minimize the cross-reactivity and avoid the nonspecific signal.

### 2.5. Application of the Dual Signal Amplification-Based Detection Method in Clinical Samples

The lungs, spleen, lymph nodes, sera, and oral samples from the 12 pigs that presented with suspected PMWS were evaluated using this method. The results showed that the samples of eight spleens and inguinal lymph nodes, six lungs, two sera, and three fecal samples were PCV2-positive, as seen in Table 2. These results were similar to those obtained by PCR assay.

PCV2 is the major etiological agent of PMWS. It is also characterized as the modulator of host immunity that induces the clinical outcome of many other pathogen infections. Thus, rapid PCV2 detection in pig herds is essential for PCV2 control. In a previous study, a PCV2 detection method was developed through PCR. However, these methods may show complexity and false positive results. 

## 3. Materials and Methods

### 3.1. Materials and Reagents

The PCV2 strain AY035820, was a gift from Prof. G. Zhang and was propagated in PK-15 cells. PRRSV, Classical swine fever virus, (CSFV), Japanese encephalitis virus (JEV), Pseudorabies virus (PRV), and Porcine parvovirus (PPV) were purchased from the China Institute of Veterinary Drug Control. Hydrogen tetrachloroaurate dydrate (HAuCl_4_·4H_2_O), glucose, sodium bicarbonate (NaHCO_3_) were obtained from Thermo Fisher Scientific (logan, OH, USA). All chemicals were guaranteed reagent-grade. Bovine serum albumin (BSA), horseradish peroxidase (HRP), and Tween-20 were purchased from Sigma-Aldrich (St. Louis, MO, USA). Biotin-tyramide (BT), Streptavidin-HRP (SA-HPR), and 3,3′, 5,5′-tetramethylbenzidine (TMB) was from Solarbio (Beijing, China). PCV2 antibodies (orb312690 and GTX133638) were purchased from Biorbyt (Wuhan, China).

### 3.2. Preparation of GNPL-Modified Microplate

A GNPL was prepared on a microplate (ELISA plate, Corning Costar, 2592, Cambridge, MA, USA) via a chemical reduction method. The specific procedure was as follows: 25 mL of a reaction solution containing 12 mM HAuCl_4_·4H_2_O, 0.5 M NaHCO_3_, and 25 mM glucose was prepared. After the solution was sufficiently dissolved, different volumes (10, 30, 60, and 90 µL) of the mixed solution were added to each well of the microplate and kept at 37 °C for 3 h. After the reaction was completed, the liquid in the well was discarded, washed three times with washing buffer, and patted dry. The morphological characteristics of the GNPL on the microplate were determined using a Hitachi S-4800 scanning electron microscope (Tokyo, Japan) at 20.0 kV.

### 3.3. Conjuated HRP and PCV2 Antibody

The HRP-labeled anti-PCV2 antibody (anti-PCV2-HRP) were prepared using sodium metaperiodate (NaIO4) by using a FastLink HRP labeling Kit (KA1551, Abnova, Taipei, Taiwan) in accordance with the manufacturer’s instructions and following the method described previously [19].

### 3.4. Procedure of the Amplified ELISA

The main procedure of this method was as follows: the indicated volume of nanogold buffer was added to each well and incubated at 37 °C for 3 h. The solution was discarded and washed with washing buffer (0.05% Tween-20 Phosphate Buffer solution, PBST) three times. Then, 100 μL of the anti-PCV2 capture antibody (10 μg/mL) in coating buffer was added to each well and incubated at 4 °C overnight. The solutions were discarded and washed three times to remove the unbound capture antibodies. Afterward, 100 μL blocking buffer (5% BSA) was injected into the wells and kept at 37 °C for 2 h, thereby blocking the nonspecific binding sites. The wells were washed again, and PCV2 in the PBS was added to the wells. The plate was kept at 37 °C for 1 h and washed three times. Later, 100 μL of the anti-HRP-PCV2 antibody solution was added into each well and incubated at 37 °C for 1 h. After the specimen was washed, 100 μL biotin-tyramine was added to the wells, kept at room temperature for 15 min, and washed three times. Then, 100 μL streptavidin-HRP was added to the wells and kept at room temperature for 20 min. The plates were washed again and incubated with 100 μL TMB for color development. After 5 min of incubation at room temperature, the enzymatic reaction was stopped with 50 μL of a stop solution (2 M H_2_SO_4_) and the reaction product was quantified at A450 (OD450) by using an automatic plate reader (Bio-Tek Instruments, Inc., Winooski, VT, USA). Data were measured in triplicate, and the mean OD450 values and standard deviation for each sample were calculated.

### 3.5. Optimization of ELISA Working Conditions

The optimal dilution of anti-PCV2, anti-PCV2-HRP, BT and streptavidin-HRP were determined in accordance with the procedure described above via checkerboard titration. In brief, anti-PCV2 was immobilized onto 96-well microplates in serial two-fold dilutions (2, 4, 6, 8, and 10 μg/mL). Anti-PCV2-HRP was diluted with carbonate buffer solution in a series of 0.1, 0.3, 0.6, 0.9, and 1.2 μg/mL. After the optimal antibody dilution was determined, the optimal TSA dilution was also defined. BT and streptavidin-HRP were added to the wells in odder at dilutions of 1, 2, 5, 10, 15 μL/mL and 1:500, 1:750, 1:000, 1:1500, 1:2000, respectively. The conditions giving the highest OD450 ratio between the positive and negative sample (*P*/*N* value) with the OD450 of the positive sample close to 1.0 were scored as the optimal working conditions of the ELISA.

### 3.6. Validation of ELISA

In this procedure, 40 PCV2-negative cell supernatant specimens were tested with the constructed method to determine the cut-off value of this assay, and the cut-off value was identified as the mean OD450 of the negative sera (X) plus two times the standard deviation (X + 2 × SD). ELISA was then validated with PCR by using the samples collected from 12 pigs with suspected PCV2 infection. The positive cell supernatants against PCV2, CSFV, JEV, PRV, and PPV were tested in accordance with the ELISA procedure to detect the specificity of ELISA.

## 4. Conclusions

In this study, a method for highly sensitive PCV2 detection was established via the first amplification of a nanogold-modified microplate and the second amplification of tyramide-accelerated HRP deposition. The method was highly sensitive, and the detection limit was as low as 0.1 TCID50. The establishment of a highly sensitive detection method would provide a simple and convenient route for epidemiological investigations and etiology eradication.

## Figures and Tables

**Table 1:** Reproductivity test.

Sample	A	B	C
Coefficient variation of interassay	5.24%	4.76%	4.25%
Coefficient variation of intra-assay	6.13%	4.86%	5.13%

**Table 2:** Detection of virus in clinical samples ^1^.

Sample Source	Number of Positive	Number of Negative
Lung	3	9
Spleen	9	3
Lymph node	10	2
Serum	1	11
Oral	4	8
Total	27	33

^1^ Clinical samples collected from a pig farm and a slaughterhouse were analyzed using the proposed method, and the results were confirmed by PCR.
